# Retraction Note: nAChRs gene expression and neuroinflammation in APPswe/PS1dE9 transgenic mouse

**DOI:** 10.1038/s41598-024-80730-6

**Published:** 2024-11-26

**Authors:** Chiara D’Angelo, Erica Costantini, Nieves Salvador, Michele Marchioni, Marta Di Nicola, Nigel H. Greig, Marcella Reale

**Affiliations:** 1 Department of Medical, Oral and Biotechnological Sciences, University “G. D’Annunzio”, Via dei Vestini 31, 66100 Chieti, Italy; 2 Department of Molecular, Cellular and Developmental Neurobiology, Instituto Cajal-CSIC, Madrid, Spain; 3 Drug Design and Development Section, Translational Gerontology Branch, Intramural Research Program, National Institute On Aging, National Institutes of Health, Baltimore, MD 21224 USA

Retraction of: *Scientific Reports* (2021) 11:9711. 10.1038/s41598-021-89139-x, published on 06 May 2021

The Editors have retracted this Article. After publication, concerns were raised regarding areas of high similarity in the green signals in Fig. 1A, B and C. The Authors have been unable to provide the underlying raw data to address these concerns.

Since Fig. 1 represents the validation of the model used in the study, the Editors no longer have confidence in the presented data.

Marcella Reale, Erica Costantini, and Michele Marchioni do not agree to this retraction. Chiara D’Angelo, Nieves Salvador, Marta Di Nicola, and Nigel H. Greig have not responded to any correspondence from the Editors about this retraction.

